# Supplementary material for: Mapping the evolution of fertility support policies in China: A content and instrumental analysis
Source: PLoS One. 2025 Oct 9;20(10):e0332137. doi: 10.1371/journal.pone.0332137 (PMC12510515; doi:10.1371/journal.pone.0332137)
Supplement: S1 Appendix — (ZIP) [file pone.0332137.s001.zip › S1 Appendix. 226 original policy documents/34-国家卫生健康委办公厅关于印发托育机构婴幼儿伤害预防指南(试行)的通知(FBM-CLI-4-351699).doc]

国家卫生健康委办公厅关于印发托育机构婴幼儿伤害预防指南(试行)的通知

**发布部门：** [国家卫生健康委员会](https://www.pkulaw.com/law/chl?Aggs.IssueDepartment=60321) [机构沿革](javascript:void(0))

**发文字号：**国卫办人口函〔2021〕19号

**发布日期：**2021.01.12

**实施日期：**2021.01.12

**时效性：** [现行有效](https://www.pkulaw.com/law/chl?Aggs.TimelinessDic=01)

**效力级别：** [部门规范性文件](https://www.pkulaw.com/law/chl?Aggs.EffectivenessDic=XE0302)

**法规类别：** [卫生综合规定](https://www.pkulaw.com/law/chl?Aggs.Category=09601)

国家卫生健康委办公厅关于印发托育机构婴幼儿伤害预防指南（试行）的通知
（国卫办人口函〔2021〕19号）

各省、自治区、直辖市及新疆生产建设兵团卫生健康委：
　　为进一步加强对托育机构的指导，提高托育机构服务质量，保障婴幼儿安全健康成长，国家卫生健康委组织编写了《托育机构婴幼儿伤害预防指南（试行）》。现印发给你们，供参考。

国家卫生健康委办公厅
2021年1月12日

托育机构婴幼儿伤害预防指南（试行）

　　为贯彻落实《[国务院办公厅关于促进3岁以下婴幼儿照护服务发展的指导意见](https://www.pkulaw.com/chl/434b24b8b5769383bdfb.html)》（国办发〔2019〕15号）精神，我委依据《托育机构设置标准（试行）》和《托育机构管理规范（试行）》（国卫人口发〔2019〕58号）、《托儿所、幼儿园建筑设计规范（2019年版）》、《儿童伤害预防与控制工作指南》等，组织编写了《托育机构婴幼儿伤害预防指南（试行）》。本指南适用于经有关部门登记、卫生健康部门备案，为3岁以下婴幼儿提供全日托、半日托、计时托、临时托等托育服务的机构。
　　伤害是儿童面临的重要健康威胁，造成了沉重的疾病负担。婴幼儿伤害的发生与其自身生理和行为特点、被照护情况、环境等诸多因素有关。常见的伤害类型包括窒息、跌倒伤、烧烫伤、溺水、中毒、异物伤害、道路交通伤害等。大量证据表明，伤害不是意外，可以预防和控制。
　　托育机构应当最大限度地保护婴幼儿的安全健康，切实做好伤害防控工作，建立伤害防控监控制度，制定伤害防控应急预案，重点开展五方面工作：第一，根据现有法律和相关规定要求，落实安全管理的主体责任，健全细化安全防护制度，认真执行各项安全措施。第二，排查并去除托育机构内环境安全隐患，提升环境安全水平。第三，规范和加强对婴幼儿的照护。第四，开展针对工作人员、家长以及幼儿的伤害预防教育和技能培训。第五，加强对工作人员的急救技能培训，配备基本的急救物资。
　　本指南主要针对窒息、跌倒伤、烧烫伤、溺水、中毒、异物伤害、道路交通伤害等3岁以下婴幼儿常见的伤害类型，为托育机构管理者和工作人员在安全管理、改善环境、加强照护等方面开展伤害预防提供技术指导和参考。
　　一、婴幼儿窒息预防
　　窒息是指呼吸道内部或外部障碍引起血液缺氧的状态。常见的婴幼儿窒息原因包括被床上用品、成人身体、塑料袋等罩住口鼻；吸入和咽下食物、小件物品、呕吐出的胃内容物等阻塞气道；绳带等绕颈造成气道狭窄；长时间停留在密闭空间导致缺氧等。
　　（一）安全管理。
　　制定和落实预防婴幼儿窒息的管理细则，主要内容包括：婴幼儿生活环境和娱乐运动设备导致窒息风险的定期排查和清除；婴幼儿睡眠、喂养照护与管理；婴幼儿服饰、玩具安全管理；工作人员预防婴幼儿窒息的安全教育和技能培训。
　　（二）改善环境。
　　1．将绳带、塑料袋、小块食物、小件物品等可造成婴幼儿绕颈或窒息的物品放在婴幼儿不能接触的位置。
　　2．使用玩具、儿童用品等前后，检查有无零件、装饰物、扣子等破损、脱落或丢失。
　　3．排除护栏、家具、娱乐运动设备中可能卡住婴幼儿头颈部的安全隐患。
　　4．在橱柜、工具房等密闭空间设置防护设施，防止婴幼儿进入。
　　（三）加强照护。
　　1．婴幼儿睡眠时，检查其口鼻是否被床上用品、衣物等覆盖，并及时清除。
　　2．不喂食易引起窒息的食物；婴幼儿进食时保持安静，避免跑跳、打闹等行为。
　　3．婴幼儿在娱乐运动设备上玩耍时，加强看护，避免拉绳、网格等造成窒息。

　　二、婴幼儿跌倒伤预防
　　跌倒伤是指一个人因倒在地面、地板或其他较低平面上的非故意事件造成的身体损伤。常见的婴幼儿跌倒伤原因包括：滑倒；从家具、楼梯或娱乐运动设备上跌落；从阳台坠楼等。婴幼儿正处于运动能力的发展过程中，跌倒较常见，托育机构应加强防护，预防婴幼儿跌倒伤。
　　（一）安全管理。
　　制定和落实预防婴幼儿跌倒伤的管理细则，主要内容包括：严格执行《托儿所、幼儿园建筑设计规范（2019年版）》相关条文；婴幼儿生活环境和娱乐运动设备跌倒伤风险的定期排查和清除；婴幼儿玩耍娱乐、上下楼、睡眠等活动的安全照护与管理；婴幼儿服饰、玩具安全管理；工作人员预防婴幼儿跌倒伤的安全教育和技能培训。
　　（二）改善环境。
　　1．地面应平整、防滑、无障碍、无尖锐突出物，并宜采用软质地坪；清除可能绊倒婴幼儿的家具、电线、玩具等物品。
　　2．楼梯处装有楼梯门，确保婴幼儿不能打开。
　　3．规范安装娱乐运动设备，设备周围地面使用软质铺装。
　　4．婴幼儿床有护栏。
　　5．在窗户、楼梯、阳台等周围不摆放可攀爬的家具或设施。
　　6．墙角、窗台、暖气罩、窗口竖边等阳角处应做成圆角，家具选择圆角或使用保护垫。
　　（三）加强照护。
　　1. 工作人员与家长沟通，为婴幼儿选择适宜活动的鞋、衣服等服饰。
　　2．为婴幼儿换尿布、衣物时，工作人员应专心看护，始终与其保持近距离，中途不能离开。
　　3．婴幼儿使用娱乐运动设备过程中或上下楼梯时，工作人员应加强看护，与其保持较近距离并确保婴幼儿在视线范围内。
　　4．婴幼儿玩耍运动前，对玩耍运动环境、设备设施进行安全性检查。

　　三、婴幼儿烧烫伤预防
　　烧烫伤是由热辐射导致的对皮肤或者其他机体组织的损伤，包括皮肤或其他组织中的部分或全部细胞因热液（烫伤）、热的固体（接触烧烫伤）、火焰（烧伤）等造成的损伤以及由放射性物质、电能、摩擦或接触化学物质造成的皮肤或其他器官组织的损伤。常见的婴幼儿烧烫伤原因包括热粥、热水等烫伤，取暖设备等烫伤，蒸汽高温等烫伤，火焰烧伤等。
　　（一）安全管理。
　　制定和落实预防婴幼儿烧烫伤的管理细则，主要内容包括：严格执行《托儿所、幼儿园建筑设计规范（2019年版）》相关条文；婴幼儿生活环境烧烫伤风险的定期排查和清除；婴幼儿进食、玩耍娱乐、洗浴清洁等活动照护与管理；婴幼儿玩具用品、电器、取暖设备安全管理；工作人员预防婴幼儿烧烫伤的安全教育和技能培训。
　　（二）改善环境。
　　1．设置热水器出水最高温度应低于45摄氏度。
　　2．设置专门区域存放热水、热饭菜、温奶器、消毒锅等物品，专用房间放置开水炉，并设置防护措施防止婴幼儿接触；使用门栏或护栏等防止婴幼儿误入厨房、浴室等可能造成烧烫伤的区域。
　　3．桌子、柜子不使用桌布等覆盖物，以避免婴幼儿拉扯桌布，热源物倾倒、坠落。
　　4．化学用品、打火机、火柴等物品专门保管并上锁；不使用有明火的蚊香驱蚊。
　　（三）加强照护。
　　1．婴幼儿饮食、盥洗前检查温度。
　　2．加热、取放热物时观察周围有无婴幼儿，避免因碰撞、泼洒造成烫伤。
　　3．安全使用暖水袋等可能造成婴幼儿烫伤的用品。

　　四、婴幼儿溺水预防
　　溺水为一个因液体进入而导致呼吸损伤的过程。常见的婴幼儿溺水地点包括：浴缸、水盆、水桶等室内设施；池塘、游泳池等室外场所。
　　（一）安全管理。
　　制定和落实预防婴幼儿溺水的管理细则，主要内容包括：婴幼儿生活环境溺水风险的定期排查和清除；婴幼儿洗浴清洁、玩耍等活动照护与管理；工作人员预防婴幼儿溺水的安全教育和技能培训。
　　（二）改善环境。
　　1．托育机构内的池塘、沟渠、井、鱼缸、鱼池、涉水景观等安装护栏、护网。
　　2．水缸、盆、桶等储水容器加盖，并避免婴幼儿进入储水容器所在区域。使用完水池、浴缸、盆、桶后及时排水。
　　（三）加强照护。
　　1．保持婴幼儿在工作人员的视线范围内，避免婴幼儿误入盥洗室、厨房、水池边等有水区域。
　　2．婴幼儿在水中或水边时，工作人员应专心看护，始终与其保持近距离，中途不能离开。

　　五、婴幼儿中毒预防
　　中毒是指因暴露于一种外源性物质造成细胞损伤或死亡而导致的伤害。常见的毒物包括：农药、药物、日用化学品、有毒植物、有毒气体等。本指南的中毒指急性中毒，不包括慢性中毒。
　　（一）安全管理。
　　制定和落实预防婴幼儿中毒的管理细则，主要内容包括：婴幼儿生活环境中毒风险的定期排查和清除；婴幼儿安全用药；工作人员预防婴幼儿中毒的安全教育和技能培训。
　　（二）改善环境。
　　1．将药物、日用化学品等存放在婴幼儿无法接触的固定位置。
　　2．规范使用消毒剂、清洁剂。
　　3．使用煤火取暖的房间应有窗户、风斗等通风结构，并保证正常工作；正确安装、使用符合标准的燃气热水器。
　　4．托育机构内不种植有毒植物，不饲养有毒动物。
　　（三）加强照护。
　　1．玩具及生活用品应安全无毒，同时工作人员要关注婴幼儿的啃咬行为，避免婴幼儿因啃咬而导致中毒。
　　2．避免有毒食物引起婴幼儿中毒，例如有毒蘑菇、未彻底加热煮熟的扁豆等。

　　六、婴幼儿异物伤害预防
　　异物伤害是指因各种因素导致异物进入体内，并对机体造成一定程度损伤，出现了各种症状和体征，如食道穿孔、气道梗阻、脑损伤等。婴幼儿异物伤害多因异物通过口、鼻、耳等进入身体造成损伤，常见的异物包括：食物、硬币、尖锐异物、电池、小磁铁、气球、玩具零件及碎片等。
　　（一）安全管理。
　　制定和落实预防婴幼儿异物伤害的管理细则，主要内容包括：婴幼儿生活环境异物伤害风险的定期排查和清除；婴幼儿饮食、玩耍等活动照护与管理；婴幼儿食物、玩具、儿童用品安全管理；工作人员预防婴幼儿异物伤害的安全教育和技能培训。
　　（二）改善环境。
　　1．将硬币、电池、小磁铁、装饰品（例如项链、皮筋、耳环等）、文具（例如笔帽、别针）等小件物品放置在婴幼儿接触不到的区域。
　　2．使用玩具、儿童用品等前后，检查有无零件、装饰物、扣子等破损、脱落或丢失。
　　3．定期检查家具、娱乐运动设备有无易掉落的零件、装饰物（例如螺丝钉、螺母等），并固定。
　　（三）加强照护。
　　1．及时收纳可能被婴幼儿放入口、鼻、耳等身体部位的小件物品。
　　2．及时制止婴幼儿把硬币、电池等小件物品放入口、鼻、耳等身体部位的行为。
　　3．选择适龄玩具，不提供含有小磁铁、小块零件的玩具。
　　4．不提供易导致异物伤害的食物，如含有鱼刺、小块骨头的食物。

　　七、婴幼儿道路交通伤害预防
　　道路交通伤害是指道路交通碰撞造成的致死或非致死性损伤。道路交通碰撞是指发生在道路上至少牵涉一辆行进中车辆的碰撞或事件。
　　（一）安全管理。
　　制定和落实预防婴幼儿道路交通伤害的管理细则，主要内容包括：托育机构车辆安全要求和管理制度，携带婴幼儿出行安全管理制度；托育机构内车辆行驶、停放安全管理制度，运输婴幼儿出行车辆驾驶员的资质要求，儿童安全座椅安全使用要求；工作人员预防婴幼儿道路交通伤害的安全教育和技能培训。
　　（二）改善环境。
　　1．托育机构内将婴幼儿活动区域与车辆行驶和停靠区域隔离。
　　2．托育机构出入口设立专门安全区域。
　　3．托育机构出入口与道路间设置隔离设施。
　　（三）加强照护。
　　1．携带婴幼儿出行时，应严格遵守道路交通法规。
　　2．携带婴幼儿出行时，密切看管并限制婴幼儿随意活动。
　　3．携带婴幼儿出行时，给婴幼儿穿戴有反光标识的衣物。
　　4．婴幼儿乘坐童车出行时，规范使用童车安全带。

　　八、其他伤害预防
　　除上述伤害类型以外，还要注意动物伤、锐器伤、钝器伤、冻伤、触电等其他类型伤害的预防控制。托育机构应针对本地区3岁以下婴幼儿实际面临的伤害问题，开展伤害防控工作，最大程度地确保婴幼儿健康安全。

　　九、婴幼儿伤害紧急处置提示
　　1.日常加强工作人员的急救知识培训，掌握基本急救技能。
　　2.发生严重婴幼儿伤害时，立即呼救并拨打120急救电话。等待救援期间，密切关注婴幼儿的生命体征，在掌握急救技能的前提下先予以现场急救。
　　3.非严重婴幼儿伤害可先自行处置，并根据伤害情况决定是否送医。
　　4.通知监护人。
　　附件：

托育机构急救物资配置建议

　　1. 消毒物品：碘伏或碘伏棉签，酒精或酒精棉片，生理盐水或生理盐水湿巾、消毒湿巾。
　　2. 包扎固定物品：纱布绷带，医用胶带，三角巾，有条件可配备自粘绷带、止血带、网状弹力绷带、不同型号夹板等。
　　3. 敷料：医用无菌纱布（大方纱、小方纱）、创可贴、干净方巾、棉签。
　　4. 器械：医用剪刀、镊子、体温计、一次性无菌手套、安全别针。
　　5. 常用药：退热药、抗生素软膏、补液盐、抗过敏药。
　　6. 其他：手电筒、急救手册、急救电话卡、紧急联系卡、急救毯、冰袋、退热贴；有条件可配备转运婴幼儿用的担架或平板。

© 北大法宝：（[www.pkulaw.com](https://www.pkulaw.com/)) 专业提供法律信息、法学知识和法律软件领域各类解决方案。北大法宝为您提供丰富的参考资料，正式引用法规条文时请与标准文本核对。
欢迎查看所有[产品和服务](https://www.pkulaw.com/helps/2.html)。法宝快讯：[如何快速找到您需要的检索结果？](https://www.pkulaw.com/helps/70.html)[法宝](https://www.pkulaw.com/helps/69.html)V6有何新特色？

扫描二维码阅读原文

**原文链接：**<https://www.pkulaw.com/chl/6421f23491cd30c2bdfb.html>
